# Atorvastatin Reduces Plasma Levels of Chemokine (CXCL10) in Patients with Crohn's Disease

**DOI:** 10.1371/journal.pone.0005263

**Published:** 2009-05-06

**Authors:** Olof Grip, Sabina Janciauskiene

**Affiliations:** 1 Division of Gastroenterology and Hepatology, Department of Clinical Sciences Malmö, Lund University, University Hospital MAS, Malmö, Sweden; 2 Chronic Inflammatory and Degenerative Diseases Research Unit, Department of Clinical Sciences Malmö, Lund University, University Hospital MAS, Malmö, Sweden; HelmholtzZentrum München, Germany

## Abstract

**Background:**

In Crohn's disease high tissue expression and serum levels of chemokines and their receptors are known to correlate with disease activity. Because statins can reduce chemokine expression in patients with coronary diseases, we wanted to test whether this can be achieved in patients with Crohn's disease.

**Methodology/Principal Findings:**

We investigated plasma levels of chemokines (CCL2, CCL4, CCL11, CCL13, CCL17, CCL22, CCL26, CXCL8, CXCL10) and endothelial cytokines (sP-selectin, sE-selectin, sICAM-3, thrombomodulin) in ten Crohn's disease patients before and after thirteen weeks' daily treatment with 80 mg atorvastatin. Of the 13 substances investigated, only CXCL10 was found to be significantly reduced (by 34%, p = 0.026) in all of the treated patients. Levels of CXCL10 correlated with C-reactive protein (r = 0.82, p<0.01).

**Conclusions/Significance:**

CXCL10 is a ligand for the CXCR3 receptor, the activation of which results in the recruitment of T lymphocytes and the perpetuation of mucosal inflammation. Hence the reduction of plasma CXCL10 levels by atorvastatin may represent a candidate for an approach to the treatment of Crohns disease in the future.

**Trial Registration:**

ClinicalTrials.gov NCT00454545

## Introduction

Over the past years, investigators from many scientific disciplines have demonstrated that the scope of chemokine function extends far beyond their chemoattractant moieties. Consequently, a growing number of reports indicate their crucial roles in a variety of diseases, among others renal diseases, rheumatoid arthritis, arteriosclerosis, multiple sclerosis and chronic inflammatory bowel diseases (IBD) [1]. Chemokines can be classified, based on functional criteria, into inflammatory and homeostatic. Typical examples of inflammatory chemokines include CCL2, CCL3, CCL4, CCL5, CCL11, CXCL8 and CXCL10. These chemokines play an important role in both innate and adaptive immunity, and are expressed by the circulating leukocytes and other cells only in response to infection, tissue damage or specific mediators, such as tumor necrosis factor, interferon-γ and microbial products [2]. In contrast, homeostatic chemokines, such as CCL19, CCL21 and CXCL12, are constitutively expressed in order to coordinate cell migration and development of the immune system [3].

It is postulated that during inflammation chemokines attract specific subsets of leukocytes according to their expression of chemokine receptors [4]. For example, in the case of T cells, the chemokines CCL17 and CCL22 are ligands for the chemokine receptor CCR4, whereas CCL1 is a ligand for CCR8 expressed on a subset of Th2 cells [5]. CXCL10 and CXCL11 are ligands for CXCR3, expressed on Th1 cells [6]. Specific chemokines and their receptors are expressed on different cell types, and therefore, relative differences in receptor distribution and affinity for specific chemokines may significantly influence which cells are ultimately attracted to and activated by the individual chemokine.

Chemokine activation requires a complex cascade of steps, including phosphorylation by multiple kinases and phosphatases, degradation of transcriptional inhibitors, translocation of transcription factors from cytoplasm to nucleus, etc. [7]. These biochemical events are potential targets for therapeutic intervention. For example, glucocorticoids interfere with nuclear factor, NF-kappaB, activation and are effective in reducing chemokine production [8]. Similarly, agents that influence cAMP levels often modulate the stimulatory signals for chemokine expression [9], [10].

Various studies indicate that statins express anti-inflammatory properties and may play a role in modulating the immune system [11]. In patients with coronary artery disease, statins (atorvastatin) were found to significantly reduce expression of chemokines (i.e., CCL3, CCL4 and CXCL8) and their receptors (i.e., CCR1 and CCR2) [12]. Statin-induced reductions in CRP have been shown in patients with and without established cardiovascular disease [13]. We have also previously shown that atorvastatin reduces plasma CRP levels and inflammation in patients with Crohn's disease (CD) [14], [15]. Several chemokines and their receptors are described to have a connection with IBD and to correlate with activity of the disease [16], [17], [18]. So far, effects of statins on the chemokine production in IBD have not been investigated. Therefore, we aimed at evaluating the effects of atorvastatin on plasma chemokine levels in CD patients. We show for the first time that high-dose oral intake of atorvastatin (80 mg per day) significantly reduces plasma CXCL10, but not the other chemokines measured. Hence blocking the production of CXCL10 by atorvastatin may represent a candidate for a new approach to the treatment of CD in the future.

## Results

### Study population

Table 1 presents the characteristics of the 10 patients who participated in the study. Their median age was 32 years (range, 23–44 y), and the median disease duration was 4.5 years.

**Table 1:** Demographic data of patients.

Age (Years)	Sex	Duration of Disease (Years)	Involvement	Smoking	IBD-related drugs
24	F	1	Ileocolonic	yes	Bud
25	F	12	Ileocecal	no	AZA
31	F	1	Ileocolonic	no	5-ASA
42	F	8	Ileocolonic	no	Pred/5-ASA
44	F	1	Oesophagus to rectum	yes	
23	M	0.5	Ileal and rectal	no	Bud
27	M	0.5	Ileocolonic	yes	
33	M	15	Ileocolonic	no	
35	M	1	Ileal	no	Bud
40	M	8	Ileal	yes	

IBD, inflammatory bowel disease; 5-ASA, 5-aminosalicylate; AZA, azathioprine; Bud, budesonide; Pred, prednisolone.

### Plasma Levels of Cytokines and Chemokines

**Figure 1:**
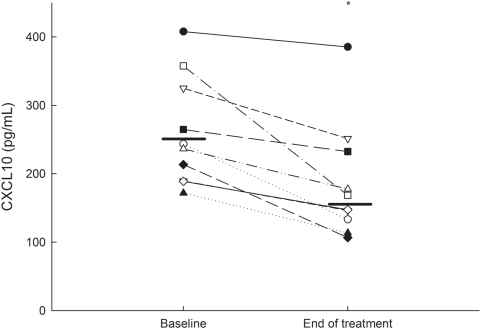
Plasma levels of CXCL10/IP-10 in ten Crohn's disease patients at baseline and after thirteen weeks' treatment with 80 mg atorvastatin daily, *P<0.05.

Table 2 and 3 show the results of the analysis of nine inflammatory chemokines and four endothelial cytokines in the patients. The top three (CXCL10, CCL4 and CCL22) in table 1 and thrombomodulin in table 2 show separately significant differences between the baselines and at the end of the treatment. Although these results are of some interest in their own right, the greatest separation was found for CXCL10 and, when corrected for multiple analyses, this was the only statistically proven difference, presented separately in figure 1. As seen in this graph all patients responded with a reduction in CXCL10 levels and the gradient did not differ between those with a higher and those with a lower CXCL10 baseline level. The mean reduction of the five patients with the highest levels was 27 percent and for the five with the lowest levels it was 31 percent. Thus, although our material is too small to subdivide into groups, our findings indicate that the effect of statin is not related to the baseline levels of CXCL10.

**Table 2:** Median plasma levels of chemokines at baseline and after 13 weeks' treatment with atorvastatin.

	Baseline (q1–q3) (pg/mL)	End of treatment (q1–q3) (pg/mL)	Δ%	Number of patients with a reduction	*P*
CXCL10/IP-10	240.1 (189.4–325.1)	158.0 (133.3–232.5)	−34	10/10	0.026
CCL4/MIP-1β	312.0 (283.1–387.3)	252.1 (219.4–283.6)	−19	9/10	0.058
CCL22/MDC	7728.2 (5755.5–9901.1)	6544.3 (3671.1–9598.1)	−15	9/10	0.490
CCL26/Eotaxin-3	43.5 (41.4–45.7)	31.8 (23.1–34.3)	−27	8/10	0.922
CCL11/Eotaxin	526.1 (404.0–723.0)	463.5 (316.0–560.7)	−12	7/10	0.512
CXCL8/IL-8	9.3 (6.2–12.3)	7.0 (4.3–9.7)	−25	7/10	0.588
CCL17/TARC	446.4 (266.1–708.8)	380.3 (211.4–537.5)	−15	7/10	0.922
CCL2/MCP-1	126.0 (99.8–127.8)	107.9 (90.9–150.8)	−14	5/10	0.922
CCL13/MCP-4	450.6 (140.7–584.2)	272.6 (117.9–693.1)	−40	4/10	0.922

*P*, corrected for multiple analyses.

**Table 3:** Median plasma levels of endothelial cytokines at baseline and after 13 weeks' treatment with atorvastatin.

	Baseline (q1–q3) (ng/mL)	End of treatment (q1–q3) (ng/mL)	Δ%	Number of patients with a reduction	*P*
Thrombomodulin	2.1 (1.9–2.2)	1.9 (1.7–2.0)	−10	8/10	0.407
sP-Selectin	243.3 (190.9–332.2)	215.3 (197.7–253.1)	−12	7/10	0.512
sICAM-3	5.3 (4.7–7.4)	4.9 (4.5–5.5)	−8	6/10	0.922
sE-Selectin	17.5 (12.6–24.7)	15.0 (8.7–20.9)	−14	5/10	0.922

*P*, corrected for multiple analyses.

### Association between plasma CXCL10 versus systemic and local markers of inflammation

To determine the relationship of CXCL10 with regard to known markers of systemic and intestinal inflammation in CD we correlated CXCL10 to the C-reactive protein (CRP) and the fecal calprotectin levels previously described in the patients [15]. At baseline the levels of CRP were 8.8 (5.7–14.4) mg/L whereas at the end of treatment the CRP levels were significantly reduced 4.9 (3.3–6.2) mg/L (p<0.01). Fecal calprotectin, a marker for intestinal inflammation, was found to be reduced after treatment in 8/10 patients [734 (251–1233) vs 384 (151–958) mg/kg], p = 0.23. Plasma CRP and CXCL10 were found to correlate before (r = 0.66, p<0.05) and after (r = 0.82, p<0.01) treatment with atorvastatin. It can be noted that after treatment the correlation was also found between CRP and calprotectin (r = 0.78, p<0.01) but not between CXCL10 and calprotectin.

As described in our previous publication, atorvastatin treatment gives a profound hypocholesterolemic effect in CD patients [15]. However, we found no relation between plasma CXCL10 levels and total cholesterol, apolipoprotein B or trigycerids, either at baseline or at the end of treatment with atorvastatin (Pearson product moment correlation calculations, p>0.05).

## Discussion

We have previously published results showing that high-dose intake of atorvastatin reduces plasma levels of CRP and clinical disease activity in CD patients [15]. Infiltration of leukocytes is a prominent histological feature of CD and the up-regulated chemokine expression correlates with increasing activity of the CD [19], [20]. On this basis we therefore hypothesized that the reduction of the CD activity achieved by the atorvastatin treatment may be related to its ability to reduce chemokine levels. The chemokines we measured have a connection with inflammatory bowel diseases, although some of them have not been shown to be elevated in plasma [16], [17], [18]. Our results show for the first time that intake of atorvastatin significantly reduces plasma levels of CXCL10 in patients with CD.

Chemokines and their receptors are molecules that may manage selective migration of particular T-cell subsets. Lymphocytes that change to IFN-γ producing Th1 effector cells express chemokine receptors such as CCR5 and CXCR3 [21]. High CXCR3 expression was originally thought to be restricted to activated T lymphocytes [22], however, later findings show that expression of CXCR3 can be detected in endothelial cells, dendritic cells, as well as in neutrophils and eosinophils within tissues dominated by Th1 [23], [24]. Both animal studies [25] and studies in CD patients [26] confirm the generally accepted assumption that CD is primarily a condition in which the Th1/Th17-type is dominant involving up-regulation of IFN-γ and IL-12 production [27].

Chemokines such as CXCL9, CXCL10 and CXCL11 all elicit their chemotactic functions by interacting with the chemokine receptor, CXCR3. CXCL10 is an immediate-early gene that is induced by IFN-γ and is produced by various cell types involved in leukocyte trafficking, such as epithelial cells [28]. The association among CXCL10, CXCR3, and Th1-dependent immunity has been observed in several models of inflammatory diseases. Therefore, there is a growing interest in the CXCL10-CXCR3 axis in inflammatory bowel disease. For instance, the up-regulated expression of chemokines, such as CCL2, CCL3, CCL4, CCL7, CCL8 and CXCL8 has been shown in mucosal biopsies from patients with CD and ulcerative colitis, and this up-regulation correlated with the disease activity [29]. As a confirmation of this, others have shown that CXCR3 and the ligands for this receptor, CXCL9 and CXCL10, are up-regulated in tissues from CD patients [30]. Similarly, serum levels of CXCL9, CXCL10 and CXCL11 were found to be significantly higher in CD patients as compared to healthy controls [31]. Studies in animal models have also shown that the leukocyte-derived CXCL10 is associated with chronic colitis, and that the blockade of CXCL10 with anti-CXCL10 antibody may abrogate the inflammatory response [32], [33]. Therefore, specific agents capable of inhibiting chemokine synthesis and secretion or blocking chemokine–chemokine receptor interaction are of particular interest in the study of patients with ulcerative colitis and CD.

Previous studies have reported a reduction of plasma chemokines (i.e CCL2, CCL4, CXCL8) in patients with cardiovascular disease treated with statins [12], [34]. Notably in our study, we observed no statistically significant changes in plasma CCL2, and other chemokine (i.e. CCL11, CCL13, CCL17, CCL22 and CCL26) levels in CD patients treated with atorvastatin. In addition, in our CD patient group atorvastatin shows no effect on adhesion molecules linked to leukocyte trafficking, such as E-selectin, P-selectin and ICAM-3, whereas in cardiovascular disease patients these adhesion molecules can be reduced by the statin therapy [35], [36], [37]. One can only speculate that the reason for these differences may reflect different effects of statins in relation to dosage, and co-treatments, and may reflect differences between the statin used, since all statins may not have the same therapeutic potential. Moreover, the effect of statins may vary between different inflammatory conditions depending on the status of inflammation and the cellular source of the chemokines and adhesion molecules. For example, some statins even have differing effects on protein expression in the same cell type: in monocytes stimulated by lipopolysaccharide, pravastatin and fluvastatin may induce the production of TNF-α [38], [39], whereas atorvastatin and simvastatin inhibit the production of TNFα [40].

Previous studies have shown that in CD patients the expression of mucosal microvasculature adhesion markers, such as CAMs, are increased [41], and a high expression of thrombomodulin, another marker of the microvasculature activation, has also been shown in CD patients [42]. There are publications showing that serum levels of E-selectin are not changed in CD whereas sP-selectin levels are dependent on disease activity [43], [44]. Thus, our findings that atorvastatin did not significantly decrease plasma levels of the soluble selectins and the thrombomodulin do not exclude the possibility that statin may affect the local expression of these proteins. On the other hand the study maybe underpowered to show less pronounced changes.

Taken together our data allow us to speculate that treatment of CD patients with atorvastatin not only lowers plasma CRP, a marker of systemic inflammation, but also specifically lowers levels of plasma chemokine, CXCL10. The reduction in CXCL10 levels after atorvastatin treatment has, to date, only been found in monocyte and epithelial cell culture models, *in vitro* [45]. Since there are no reports regarding the effects of statins on this chemokine in patients with inflammatory bowel diseases, we are not able to conclude whether our finding is specifically related to CD patients.

There are several possible pathways through which statins could reduce chemokine synthesis and release. For example, by inhibiting HMG-CoA reductase, statins can block the synthesis of important isoprenoid intermediates, which are necessary for the post-translational lipid modification (prenyalation) of a variety of intracellular signaling molecules [46]. In particular, the inhibition of small guanosine triphosphate (GTP)-binding proteins Rho, Ras, and Rac, whose proper localisation and function in the membrane are dependent upon isoprenylation, plays an important role in signal transduction pathways that regulate cell proliferation, cell differentiation, vesicular transport and apoptosis [47]. Thus, the inhibitory effect on chemokine-induced migration by statins can be explained by these mechanisms since both the MAPK cascade, the NF-κB and the JNK pathways, which regulate expression and release of chemokines [48], are activated by Rho-GTPases [49], [50], [51].

Although chronic inflammatory diseases, including IBD, are associated with increased expression of specific chemokines and their receptors, the role these chemokines play in disease severity, susceptibility and progression is not certain. Furthermore, the analysis of chemokine levels in plasma may reflect the general inflammatory status but not the event at the specific site, i.e. the intestine. Whether the CXCL10 we measure in the plasma can promote specific T-cell migration into the gut remains to be determined. Yet the relevance of plasma CXCL10 levels in our study is supported by its close correlation to CRP, which is an established marker of intestinal inflammation in CD patients [52].

In conclusion we show, that atorvastatin significantly decreases plasma levels of CXCL10 in CD patients but the question whether this is a specific effect is still open for discussion. CXCL10 is an important ligand for the CXCR3 receptor on lymphocytic cells, the activation of which may result in the recruitment of T lymphocytes and the perpetuation of chronic mucosal inflammation. Thus we believe that our finding describes a new, important *in vivo* function of atorvastatin and also provides further support for the use of statins in therapy for patients with CD.

## Materials and Methods

### Study Participants

The patients and the design of the clinical trial have previously been described [15]. In short, ten patients (5 women and 5 men) with a confirmed diagnosis of Crohn's disease were given a supplementary treatment of 80 mg atorvastatin (Pfizer, Sollentuna, Sweden) once daily. All baseline drugs were kept unchanged. Plasma samples were taken prior to treatment and after 13 weeks' treatment. Analysis was done at the termination of the treatment and plasma was stored during the study at −20°C. The study was approved by the Medical Products Agency, the Regional Ethical Review Board and was performed in accordance with good clinical practice (monitored by RSKC, Region Skåne). Written informed consent was obtained from all participants. The study was registered at ClinicalTrials.gov: NCT00454545. We would like to notify, that studies using statins therapy on CD patients have not been performed previously and we had no reference, and therefore, for ethical reasons we did not want to start treatment on a large number of CD patients. We wanted first to know that CD patients, who were willing to participate in the study, were getting the right drug at the right dose, and tolerated this drug well. The patient flowchart and the protocol for this trail is available as supporting information; see Flowchart S1 and Protocol S1.

### Cytokine Multiplex Analysis

Plasma levels of chemokines and vascular cytokines were assessed by MesoScale technology, which is a multiplex type of ELISA technology that relies on electrochemiluminescence, according to the manufacturer's protocol. Briefly, specific antibodies that coat a working electrode at the bottom of the well capture the molecule of interest. A second antibody labelled with a SULFO-TAG™ binds the molecule of interest. The SULFO-TAG™ emits light upon electrochemical stimulation when a current is applied between the counter electrode and the working electrode, and is registered by a SECTORT™ Imager. Detection limits, LODs (Limits Of Detection) are defined as 2.5× the standard deviation above the background as calculated by MesoScale Discovery®: CCL2/MCP-1, 3.3 pg/mL; CCL4/MIP-1β, 14 pg/mL; CCL11/Eotaxin, 10 pg/mL; CCL13/MCP-4, 19 pg/mL; CCL17/TARC, 8 pg/mL; CCL22/MDC, 234 pg/mL; CCL26/Eotaxin-3, 14 pg/mL; CXCL8/IL-8, 0.25 pg/mL; CXCL10/IP-10, 64 pg/mL. The LODs for the vascular cytokines are: sP-selectin, 0.01 ng/mL; sE-selectin, 0.02 ng/mL; sICAM-3, 0.05 ng/mL; thrombomodulin, 0.08 ng/mL.

### Statistical analysis

Assay results are expressed as medians with the interquartile ranges (q1,q3) specified in the tables. Pair-wise comparisons were done by the Wilcoxon signed rank test between baselines vs. after 13 weeks' treatment using SigmaStat 3.1 software (Systat Software Gmbh, Germany). Corrections for multiple analyses were done by the Benjamini and Hochberg false discovery rate. Correlation analyses were done by the Pearson product moment. A *P*-value<0.05 was regarded as significant.

## Supporting Information

Flowchart S1 Consort Flowchart
(0.03 MB DOC)

Protocol S1 (0.17 MB DOC)

## References

[1] Allen SJ, Crown SE, Handel TM (2007). Chemokine: receptor structure, interactions, and antagonism.. Annu Rev Immunol.

[2] Sallusto F, Mackay CR, Lanzavecchia A (2000). The role of chemokine receptors in primary, effector, and memory immune responses.. Annu Rev Immunol.

[3] Moser B, Willimann K (2004). Chemokines: role in inflammation and immune surveillance.. Ann Rheum Dis.

[4] Luster AD (2002). The role of chemokines in linking innate and adaptive immunity.. Curr Opin Immunol.

[5] D'Ambrosio D, Iellem A, Bonecchi R, Mazzeo D, Sozzani S (1998). Selective up-regulation of chemokine receptors CCR4 and CCR8 upon activation of polarized human type 2 Th cells.. J Immunol.

[6] Sallusto F, Lenig D, Mackay CR, Lanzavecchia A (1998). Flexible programs of chemokine receptor expression on human polarized T helper 1 and 2 lymphocytes.. J Exp Med.

[7] Baggiolini M (1998). Chemokines and leukocyte traffic.. Nature.

[8] Barnes PJ (1997). Nuclear factor-kappa B.. Int J Biochem Cell Biol.

[9] Mantovani A (1999). The chemokine system: redundancy for robust outputs.. Immunol Today.

[10] Heystek HC, Thierry AC, Soulard P, Moulon C (2003). Phosphodiesterase 4 inhibitors reduce human dendritic cell inflammatory cytokine production and Th1-polarizing capacity.. Int Immunol.

[11] Jain MK, Ridker PM (2005). Anti-inflammatory effects of statins: clinical evidence and basic mechanisms.. Nat Rev Drug Discov.

[12] Waehre T, Damas JK, Gullestad L, Holm AM, Pedersen TR (2003). Hydroxymethylglutaryl coenzyme a reductase inhibitors down-regulate chemokines and chemokine receptors in patients with coronary artery disease.. J Am Coll Cardiol.

[13] Albert MA, Danielson E, Rifai N, Ridker PM (2001). Effect of statin therapy on C-reactive protein levels: the pravastatin inflammation/CRP evaluation (PRINCE): a randomized trial and cohort study.. Jama.

[14] Grip O, Janciauskiene S, Lindgren S (2004). Circulating monocytes and plasma inflammatory biomarkers in active Crohn's disease: elevated oxidized low-density lipoprotein and the anti-inflammatory effect of atorvastatin.. Inflamm Bowel Dis.

[15] Grip O, Janciauskiene S, Bredberg A (2008). Use of atorvastatin as an anti-inflammatory treatment in Crohn's disease.. Br J Pharmacol.

[16] Chen W, Paulus B, Shu D, Wilson, Chadwick V (2001). Increased serum levels of eotaxin in patients with inflammatory bowel disease.. Scand J Gastroenterol.

[17] Jugde F, Alizadeh M, Boissier C, Chantry D, Siproudhis L (2001). Quantitation of chemokines (MDC, TARC) expression in mucosa from Crohn's disease and ulcerative colitis.. Eur Cytokine Netw.

[18] Uguccioni M, Gionchetti P, Robbiani DF, Rizzello F, Peruzzo S (1999). Increased expression of IP-10, IL-8, MCP-1, and MCP-3 in ulcerative colitis.. Am J Pathol.

[19] Baumgart DC, Carding SR (2007). Inflammatory bowel disease: cause and immunobiology.. Lancet.

[20] Zhong W, Kolls JK, Chen H, McAllister F, Oliver PD (2008). Chemokines orchestrate leukocyte trafficking in inflammatory bowel disease.. Front Biosci.

[21] Qin S, Rottman JB, Myers P, Kassam N, Weinblatt M (1998). The chemokine receptors CXCR3 and CCR5 mark subsets of T cells associated with certain inflammatory reactions.. J Clin Invest.

[22] Loetscher M, Gerber B, Loetscher P, Jones SA, Piali L (1996). Chemokine receptor specific for IP10 and mig: structure, function, and expression in activated T-lymphocytes.. J Exp Med.

[23] Gasperini S, Marchi M, Calzetti F, Laudanna C, Vicentini L (1999). Gene expression and production of the monokine induced by IFN-gamma (MIG), IFN-inducible T cell alpha chemoattractant (I-TAC), and IFN-gamma-inducible protein-10 (IP-10) chemokines by human neutrophils.. J Immunol.

[24] Garcia-Lopez MA, Sanchez-Madrid F, Rodriguez-Frade JM, Mellado M, Acevedo A (2001). CXCR3 chemokine receptor distribution in normal and inflamed tissues: expression on activated lymphocytes, endothelial cells, and dendritic cells.. Lab Invest.

[25] Iqbal N, Oliver JR, Wagner FH, Lazenby AS, Elson CO (2002). T helper 1 and T helper 2 cells are pathogenic in an antigen-specific model of colitis.. J Exp Med.

[26] Schmidt C, Giese T, Ludwig B, Mueller-Molaian I, Marth T (2005). Expression of interleukin-12-related cytokine transcripts in inflammatory bowel disease: elevated interleukin-23p19 and interleukin-27p28 in Crohn's disease but not in ulcerative colitis.. Inflamm Bowel Dis.

[27] Kobayashi T, Okamoto S, Hisamatsu T, Kamada N, Chinen H (2008). IL-23 differentially regulates the Th1/Th17 balance in ulcerative colitis and Crohn's disease.. Gut.

[28] Luster AD, Ravetch JV (1987). Biochemical characterization of a gamma interferon-inducible cytokine (IP-10).. J Exp Med.

[29] Banks C, Bateman A, Payne R, Johnson P, Sheron N (2003). Chemokine expression in IBD. Mucosal chemokine expression is unselectively increased in both ulcerative colitis and Crohn's disease.. J Pathol.

[30] Dwinell MB, Lugering N, Eckmann L, Kagnoff MF (2001). Regulated production of interferon-inducible T-cell chemoattractants by human intestinal epithelial cells.. Gastroenterology.

[31] Singh UP, Venkataraman C, Singh R, Lillard JW (2007). CXCR3 axis: role in inflammatory bowel disease and its therapeutic implication.. Endocr Metab Immune Disord Drug Targets.

[32] Singh UP, Singh S, Taub DD, Lillard JW (2003). Inhibition of IFN-gamma-inducible protein-10 abrogates colitis in IL-10−/− mice.. J Immunol.

[33] Hyun JG, Lee G, Brown JB, Grimm GR, Tang Y (2005). Anti-interferon-inducible chemokine, CXCL10, reduces colitis by impairing T helper-1 induction and recruitment in mice.. Inflamm Bowel Dis.

[34] Blanco-Colio LM, Martin-Ventura JL, de Teresa E, Farsang C, Gaw A (2007). Elevated ICAM-1 and MCP-1 plasma levels in subjects at high cardiovascular risk are diminished by atorvastatin treatment. Atorvastatin on Inflammatory Markers study: a substudy of Achieve Cholesterol Targets Fast with Atorvastatin Stratified Titration.. Am Heart J.

[35] Bolewski A, Lipiecki J, Plewa R, Burchardt P, Siminiak T (2008). The effect of atorvastatin treatment on lipid profile and adhesion molecule levels in hypercholesterolemic patients: relation to low-density lipoprotein receptor gene polymorphism.. Cardiology.

[36] Kushiya F, Wada H, Ooi K, Sakurai Y, Sakaguchi A (2005). Effects of atorvastatin on serum lipids, lipoproteins, and hemostasis.. Am J Hematol.

[37] Rauch U, Osende JI, Chesebro JH, Fuster V, Vorchheimer DA (2000). Statins and cardiovascular diseases: the multiple effects of lipid-lowering therapy by statins.. Atherosclerosis.

[38] Rosenson RS, Tangney CC, Casey LC (1999). Inhibition of proinflammatory cytokine production by pravastatin.. Lancet.

[39] Takahashi HK, Mori S, Iwagaki H, Yoshino T, Tanaka N (2005). Differential effect of LFA703, pravastatin, and fluvastatin on production of IL-18 and expression of ICAM-1 and CD40 in human monocytes.. J Leukoc Biol.

[40] Wagner AH, Schwabe O, Hecker M (2002). Atorvastatin inhibition of cytokine-inducible nitric oxide synthase expression in native endothelial cells in situ.. Br J Pharmacol.

[41] Sanders DS (2005). Mucosal integrity and barrier function in the pathogenesis of early lesions in Crohn's disease.. J Clin Pathol.

[42] Scaldaferri F, Sans M, Vetrano S, Graziani C, De Cristofaro R (2007). Crucial role of the protein C pathway in governing microvascular inflammation in inflammatory bowel disease.. J Clin Invest.

[43] Andoh A, Tsujikawa T, Hata K, Araki Y, Kitoh K (2005). Elevated circulating platelet-derived microparticles in patients with active inflammatory bowel disease.. Am J Gastroenterol.

[44] Goke M, Hoffmann JC, Evers J, Kruger H, Manns MP (1997). Elevated serum concentrations of soluble selectin and immunoglobulin type adhesion molecules in patients with inflammatory bowel disease.. J Gastroenterol.

[45] Ortego M, Bustos C, Hernandez-Presa MA, Tunon J, Diaz C (1999). Atorvastatin reduces NF-kappaB activation and chemokine expression in vascular smooth muscle cells and mononuclear cells.. Atherosclerosis.

[46] Liao JK (2002). Isoprenoids as mediators of the biological effects of statins.. J Clin Invest.

[47] Zhang FL, Casey PJ (1996). Protein prenylation: molecular mechanisms and functional consequences.. Annu Rev Biochem.

[48] Wong CK, Li PW, Lam CW (2007). Intracellular JNK, p38 MAPK and NF-kappaB regulate IL-25 induced release of cytokines and chemokines from costimulated T helper lymphocytes.. Immunol Lett.

[49] Cammarano MS, Minden A (2001). Dbl and the Rho GTPases activate NF kappa B by I kappa B kinase (IKK)-dependent and IKK-independent pathways.. J Biol Chem.

[50] Marinissen MJ, Chiariello M, Gutkind JS (2001). Regulation of gene expression by the small GTPase Rho through the ERK6 (p38 gamma) MAP kinase pathway.. Genes Dev.

[51] Marinissen MJ, Chiariello M, Tanos T, Bernard O, Narumiya S (2004). The small GTP-binding protein RhoA regulates c-jun by a ROCK-JNK signaling axis.. Mol Cell.

[52] Solem CA, Loftus EV, Tremaine WJ, Harmsen WS, Zinsmeister AR (2005). Correlation of C-reactive protein with clinical, endoscopic, histologic, and radiographic activity in inflammatory bowel disease.. Inflamm Bowel Dis.

