# Supplementary material for: Atorvastatin Reduces Plasma Levels of Chemokine (CXCL10) in Patients with Crohn's Disease
Source: PLoS One. 2009 May 6;4(5):e5263. doi: 10.1371/journal.pone.0005263 (PMC2674206; doi:10.1371/journal.pone.0005263)
Supplement: Flowchart S1 — Consort Flowchart (0.03 MB DOC) [file pone.0005263.s001.doc]

**3** patients stopped treatment due to side-effects within the first 4 study weeks

**13** patients were enrolled in the study

**4** patients did not fulfill inclusion criteria

**17** patients were screened for the study

**10** patients were treated 13 weeks with atorvastatin
