# Supplementary material for: Atorvastatin Reduces Plasma Levels of Chemokine (CXCL10) in Patients with Crohn's Disease
Source: PLoS One. 2009 May 6;4(5):e5263. doi: 10.1371/journal.pone.0005263 (PMC2674206; doi:10.1371/journal.pone.0005263)
Supplement: Protocol S1 — (0.17 MB DOC) [file pone.0005263.s002.doc]

Datum: 060911

Version nr: 03

EudraCT nr: 2005-000496-17

Studieprotokoll

Studie ID/nr: 010

Studietitel:

Atorvastatin vid måttligt aktiv Crohns sjukdom

Författare/ansvarig prövare: Olof Grip

Namnteckning:………………………….

# Innehållsförteckning

Innehållsförteckning [2](#__RefHeading___Toc145832329)

1 Sammanfattning/Synopsis [5](#__RefHeading___Toc145832330)

2 Flödesschema [5](#__RefHeading___Toc145832331)

3 Introduktion/Bakgrund [5](#__RefHeading___Toc145832332)

4. Målsättning/”Objective(s)” [7](#__RefHeading___Toc145832333)

4.1 Primär frågeställning [7](#__RefHeading___Toc145832334)

4.2 Sekundär frågeställning [7](#__RefHeading___Toc145832335)

4.3 ”Endpoints” [7](#__RefHeading___Toc145832336)

5 Studiedesign [7](#__RefHeading___Toc145832337)

5.1 Typ av studie [7](#__RefHeading___Toc145832338)

5.2 Val av studiedesign [8](#__RefHeading___Toc145832339)

5.3 Val av behandling [8](#__RefHeading___Toc145832340)

6. Studiepopulation [8](#__RefHeading___Toc145832341)

6.1 Antal patienter i studien [8](#__RefHeading___Toc145832342)

6.2 Inklusionskriterier [8](#__RefHeading___Toc145832343)

6.3 Exklusionskriterier [8](#__RefHeading___Toc145832344)

6.4 ”Withdrawal criteria” [9](#__RefHeading___Toc145832345)

7 Tidsplanering [9](#__RefHeading___Toc145832346)

8 Metodbeskrivning [9](#__RefHeading___Toc145832347)

8.1 Tillvägagångssätt [9](#__RefHeading___Toc145832348)

8.1.1 Besök 1 (Screening, v. 0) [10](#__RefHeading___Toc145832349)

8.1.2 Besök 2 (Enrollment, v.2) [10](#__RefHeading___Toc145832350)

8.1.3 Besök 3 (v.6) [10](#__RefHeading___Toc145832351)

8.1.4 Besök 4 (v.15) [10](#__RefHeading___Toc145832352)

8.1.5 Besök 5 (Avslutande besök, v.23) [10](#__RefHeading___Toc145832353)

8.2 Effektvariabler [11](#__RefHeading___Toc145832354)

8.2.1 Blodprov [11](#__RefHeading___Toc145832355)

8.2.2 Calprotektin [11](#__RefHeading___Toc145832356)

8.2.3 Crohn’s Disease Activity Index (CDAI) [11](#__RefHeading___Toc145832357)

8.3 Säkerhetsvariabler [11](#__RefHeading___Toc145832358)

8.3.1 Oönskade händelser (Adverse Events=AE) [11](#__RefHeading___Toc145832359)

9 Samtidig medicinering [11](#__RefHeading___Toc145832360)

10 Samtidiga sjukdomar [12](#__RefHeading___Toc145832361)

11 Studiematerial [12](#__RefHeading___Toc145832362)

11.1 Läkemedel [12](#__RefHeading___Toc145832363)

11.2 Dosering/behandlingsregim [12](#__RefHeading___Toc145832364)

11.3 Förpackning och förvaring av studieläkemedel [12](#__RefHeading___Toc145832365)

11.4 Läkemedelshantering (”Drug Accountability”) [12](#__RefHeading___Toc145832366)

11.5 Randomisering och blindning [12](#__RefHeading___Toc145832367)

11.6 ”Compliance” [12](#__RefHeading___Toc145832368)

12 ”Adverse Events” (oönskade händelser) [13](#__RefHeading___Toc145832369)

12.1 Oönskade händelser (AE) [13](#__RefHeading___Toc145832370)

12.2 ”Serious Adverse Events” [13](#__RefHeading___Toc145832371)

12.3 “Suspected Unexpected Serious Adverse Reaction” [13](#__RefHeading___Toc145832372)

12.4 Definitioner [13](#__RefHeading___Toc145832373)

12.4.1 Svårighetsgrad [13](#__RefHeading___Toc145832374)

12.4.2 Relation till studieläkemedel [14](#__RefHeading___Toc145832375)

12.5 Rapportering av oönskade händelser [14](#__RefHeading___Toc145832376)

12.5.1 Rapportering av SAE - allvarliga oönskade händelser [14](#__RefHeading___Toc145832377)

12.5.2 Rapportering av SUSAR – misstänkta allvarliga oönskade reaktioner [14](#__RefHeading___Toc145832378)

13 Patientformulär (CRF= ”Case Report Form”) [14](#__RefHeading___Toc145832379)

13.1 Andra patientformulär [15](#__RefHeading___Toc145832380)

14 Monitorering [15](#__RefHeading___Toc145832381)

14.1 Källdata [16](#__RefHeading___Toc145832382)

15 Datainsamling och databearbetning [16](#__RefHeading___Toc145832383)

16 Etiska överväganden [16](#__RefHeading___Toc145832384)

16.1 Medgivande [16](#__RefHeading___Toc145832385)

16.2 Regional etik prövningsnämnd (REPN) [17](#__RefHeading___Toc145832386)

17 Läkemedelsverket (LV) [17](#__RefHeading___Toc145832387)

18 Avbrytande av studien [17](#__RefHeading___Toc145832388)

19 Statistiska överväganden [17](#__RefHeading___Toc145832389)

20 Patientförsäkring [18](#__RefHeading___Toc145832390)

21 Rapport och publicering [18](#__RefHeading___Toc145832391)

22 Förvaring/Arkivering [18](#__RefHeading___Toc145832392)

Referenslista [18](#__RefHeading___Toc145832393)

Bilagor:

Patientdagbok

Patientinformation & samtycke

Namn och adresslista för studieteam och monitor

Lista över använda förkortningar:

AE= ”Adverse Event”= Oönskad händelse.

CRF= “Case Report Form”= Patientformulär.

GCP= ”Good Clinical Practice”

ICH= ”International Conference on Harmonisation”

ITT= ”Intention-to-treat”= Inkluderar all data från alla patienter/försökspersoner som deltagit i studien.

LV= Läkemedelsverket.

LVFS= Läkemedelsverkets författningssamling

PP= ”Per Protokoll” analys = Inkluderar endast data som överensstämmer med det som är angivet i studieprotokollet.

REPN= Regional etikprövningsnämnd.

SAE= ”Serious Adverse Event”= Allvarlig oönskad händelse.

SUSAR= “Suspected Unexpexted Serious Adverse Reaction”

PUL= Personuppgiftslagen

# 1 Sammanfattning/Synopsis

Studien planeras som en öppen pilotstudie för att inhämta information avseende effekten av atorvastatin vid Crohns sjukdom. Studien är lokaliserad till ett centra med en forskningshuvudman. Syftet med studien är att efterforska huruvida atorvastatin (Lipitor®) har en kliniskt immunmodulerande effekt på patienter med måttligt aktiv Crohns sjukdom. Tidigare studier saknas. Patienter med Crohns sjukdom saknar bra underhållsbehandling, varför det är av stor vikt att få fram alternativ till denna svårbehandlade patientgrupp.

Studieläkemedlet är välbeprövat vid hyperkolesterolemi och en av de mest förskrivna läkemedel globalt sett med relativt beskedliga biverkningar. Publicerad data finns att läkemedlet har antiinflammatoriska egenskaper på patienter med annan kronisk inflammation såsom vid reumatoid artrit och multipel skleros. Studien vill göras för att kunna motivera en senare placebokontrollerad nationell studie.

Till 12 patienter med måttligt aktiv Crohns sjukdom erbjuds tilläggsbehandling med 80 mg atorvastatin dagligen under 3 månader. Patienterna rekryteras från mottagningen för medicinsk gastroenterologi och hepatologi, medicinkliniken, UMAS.

Inflammationsmarkörer i blodet kontrolleras innan behandlingsstart samt under behandling. De markörer som kommer att bedömas är CRP, MCP-1, TNF-alfa samt immunfenotypen av cirkulerande monocyter med hjälp av flödescytometri. Den kliniska aktiviteten kommer att bedömas med hjälp av ett aktivitetsindex, CDAI. Inflammationen i tarmen kommer att bedömas med hjälp av analys av calprotektin i faeces innan och under behandling.

I studien ingår 5 klinikbesök där patienten för dagbok veckan innan vardera besök. Den totala studietiden är 23 veckor. Studien kommer att utföras under 2006-2007.

# 2 Flödesschema

| **Besök nr:** | **1** | **2** | **3** | **4** | **5** |
| --- | --- | --- | --- | --- | --- |
| **Vecka nr:** | **0** | **2** | **6** | **15** | **23** |
| Informerat samtycke | X |  |  |  |  |
| Sjukdomshistoria | X |  |  |  |  |
| Fysisk undersökning | X |  |  | X | X |
| Inkl/exklusionskriterier | X | X |  |  |  |
| Faecesodling | X |  |  |  |  |
| Blodprover (rutin) | X |  | X | X | X |
| Calprotektin i faeces |  | X |  | X |  |
| Blodprov för flödescytometri | X |  | X | X | X |
| Oönskade händelser (AE) |  | X | X | X | X |
| CDAI |  | X | X | X | X |
| Utlämnande av läkemedel |  | X |  |  |  |

# 3 Introduktion/Bakgrund

Prevalensen i Sverige av patienter med Crohns sjukdom uppskattas till 200 per 100 000 invånare, med incidenstopp i åldrarna 20-30 år, vilket innebär att patienterna kommer att leva med sjukdomen större delen av sitt liv, med upprepade kontakter med sjukvården. Inflammationen är kronisk, med aktiva skov där genesen är okänd. Symptomen domineras av diarré, ofta blodtillblandad, viktnedgång pga. malabsorption och malnutrition och ibland feber. I dagsläget finns ingen botande behandling utan istället inriktas terapin på att försöka dämpa inflammationen. I första hand farmakologiskt, men i resistenta fall kan det bli nödvändigt med kirurgisk resektion av det inflammerade området.

Sjukdomen tros vara orsakad av en felriktad och ständigt pågående aktivering av immunsystemet i tarmväggen i närvaro av en normal tarmflora. Det här felaktiga immunsvaret är troligen orsakad av defekter i både tarmepitelets barriärfunktion och i själva immunsystemet. Det är dock fortfarande oklart om immunsystemet är aktiverat som ett resultat av inneboende defekter, antigen en konstitutiv aktivering eller pga icke-fungerande nedregleringsmekanismer, eller pga kontinuerlig stimulering som ett resultat av förändringar i epitelets mukosabarriär [1]. I den här processen bidrar de immunkompetenta monocyterna/makrofagerna till och vidmakthåller den kroniska inflammationen genom att fungera som både regulatoriska och effektor celler.

Dagens behandling som används vid inflammatorisk tarmsjukdom är ej optimal. Både steroider, azatioprin och metotrexat som idag är etablerade behandlingar vid Crohns sjukdom har bred immundämpande verkan med ett flertal oönskade effekter. Hos patienter med aktiv Crohns sjukdom induceras en remission med adekvata doser av kortison enbart i 60-70% av fallen, och av dessa återfaller flera i aktiv sjukdom vid den successiva dosminskningen av kortisonet. Olyckligtvis har ungefär en tredjedel av alla patienter med Crohns sjukdom en aktiv form av sjukdomen trots medicinsk behandling och mer än 70% kommer under sin livstid att genomgå kirurgi pga. stenoser, perforationer, fistlar eller refraktär sjukdom. Vi har erhållit en djupare förståelse om de mekanismer som ligger till grund för utvecklandet och fortskidandet av de immunsvar som ses vid inflammatorisk tarmsjukdom. På basis av de data vi har idag är det möjligt att en intervenering på nivån för monocytrekrytering, aktivering och/eller differentiering kan komplettera både den existerande terapin och den som håller på att utvecklas. En sådan intervention kan uppnås genom att minska rekryteringen av monocyter till inflammerad tarmvävnad eller modulering av de signalvägar som ligger till grund för syntesen och frisättandet av inflammatoriska mediatorer från dessa immunkompetenta celler.

Statiner har visat sig besitta antiinflammatoriska egenskaper vilka är väletablerade i experimentella studier [2,3]. Statiner är hämmare av enzymet HMG-CoA reduktas och är potenta läkemedel som påverkar fettomsättningen och sannolikt även har effekter på immunsystemet. HMG-CoA reduktas hämmar bildandet av mevalonsyra, som förutom att bilda kolesterol, även är förstadium till ett flertal andra substanser som har betydelse för normal cellaktivitet. Då vi behandlade monocyter tagna från patienter med aktiv Crohns sjukdom i cellodlingsförsök med statinen atorvastatin minskade utsöndringen av MCP-1 och TNF-α [4]. Vidare fann vi att då fettomsättning i monocyter nedreglerades med statinerna pravastatin eller atorvastatin minskade utsöndringen av inflammatoriska molekyler, så som MCP-1, TNF-α, MMP-9 [4, 5]. Dessutom minskade syrgaskonsumtion i dessa celler tydande på minskat bildande av skadliga fria syrgasradikaler. Slutligen fann vi att atorvastatin genom att reglera intracellulär fettomsättning aktiverar transkriptionsfaktorn PPAR-γ i monocyter [6]. Påverkan på den intracellulära fettomsättningen är en möjlig förklaringsmekanism till statiners inflammationsdämpande egenskaper. Det har nu även kommit signaler på att statiner kan fungera som antiinflammatorisk behandling vid andra kroniskt inflammatoriska sjukdommar förutom ateroskleros. Det har visat sig att behandling med statiner troligen kan minska den kliniska inflammationen både vid rematoid artrit [7] och multipel skleros [8].

Upptäckten och introduktionen av potenta, immunomodulerande alternativ som saknar oönskade biverkningar, skulle vara ett stort kliniskt genombrott i behandlingen av den svårbehandlade patientgrupp som inflammatorisk tarmsjukdom är. Trots att vi får lov att vara försiktiga att extrapolera våra tidigare resultat till en bredare klinisk situation, indikerar de om en ny möjlighet att se på behandlingen av kronisk inflammation som vidmakthålls via monocytrekrytering, genom att modulera den intracellulära lipidhomeostasen. Därutöver finns det en stor klinisk dokumentation av statiner, då de är brett använda och har visat sig ha få sidoeffekter [9], vilket gör dem till lämpliga kandidater för underhållsbehandling.

# 4. Målsättning/”Objective(s)”

## 4.1 Primär frågeställning

Undersöka hurvida statiner har en klinisk immunmodulerande effekt vid Crohns sjukdom. Den primära frågeställningen är bedömning av inflammationsgraden i blodet före och under behandling.

## 4.2 Sekundär frågeställning

Sekundär klinisk frågeställning är förändringar i kliniskt aktivitesindex, Crohns disease activity index, CDAI [10], samt inflammationsgraden i tarmslemhinnan.

## 4.3 ”Endpoints”

Primära effektparametrar. Patienterna kommer att bedömmas med avseende på inflammations markörer vecka 0 och 2 jämfört mot vecka 15. De markörer som kommer att mätas i plasma är C-reaktivt protein, TNF-alfa och MCP-1. Förändringar i immunfenotypen av cirkulerande monocyter kommer att karaktäriseras med hjälp av flödescytometri. Inflammationsgraden i tarmen kommer att bedömmas med hjälp av koloskopi.

Sekundär effektparameter är förändringar i kliniskt aktivitesindex, Crohns disease activity index (CDAI).

# 5 Studiedesign

## 5.1 Typ av studie

Studien är upplagd som en nationell singelcenter öppen, utan kontrollgrupp. Studiens längd är 23 veckor, bestående av behandling under 3 månader följt av 8 veckors uppföljning.

## 5.2 Val av studiedesign

Inga studier har tidigare utförts på patienter med Crohns sjukdom där man använt statiner som en antiinflammatorisk behandling. Syftet är att klarlägga huruvida atorvastatin kan påverka inflammationsgraden kliniskt, detta för att senare kunna motivera en nationell multicenter, placebo-kontrollerad studie.

## 5.3 Val av behandling

Patienter med säkerställd Crohns sjukdom kommer att erbjudas deltagnde i studien där de erbjuds behandling med atorvastatin 80 mg peroralt dagligen som tillägg till tidigare medicinering. Det finns data som tyder på en dos respons-effekt av statiner, där högre dosering har visat sig ha en mer uttalad sänkning av inflammationsmarkören CRP. Åttio mg atorvastatin är den högsta dos som har visat sig tolereras väl i kliniska studier för behandling av hyperlipidemi.

# 6. Studiepopulation

## 6.1 Antal patienter i studien

Tolv patienter kommer att inkluderas. I en pilotstudie rekomemenderas cirka 8 patienter, men då vi räknar med ett visst bortfall har vi valt att inkludera ytterligare 4 patienter för att ha 8 fullt utvärderbara enligt protokollet.

Studien kommer att utföras vid sektionen för gastroenterologi och hepatologi, medicinkliniken, Universitetssjukhuset MAS, Malmö.

## Inklusionskriterier

- Patienter med konsterad Crohns sjukdom.
- Patienten har givit sitt samtycke till att delta i studien.
- C-reaktivt protein >2 mg/ml i plasma eller endoskopisk bild vid koloskopi på aktiv inflammation.
- Frånvaro av infektion.
- Ålder mellan 18-65 år.
- B-hemoglobin > 100 g/L, P-albumin > 25 g/L.

## 6.3 Exklusionskriterier

- Allvarligt skov som definieras med CDAI > 450.
- Pågående lipidsänkande behandling.
- Tidigare fått biverkningar på statinbehandling.
- Prednisolondos högre än 15 mg/dag, eller dosändring senare än 4 veckor före besök 1.
- Klinisk signifikant njursjukdom eller leversjukdom; aspartataminotransferas, alaninaminotransferas, eller kreatininkinas högre än två ggr övre referensen för normalvärdet.
- Tidigare deltagande i studien
- Graviditet, amning eller planerad graviditet. För kvinnliga deltagare ska adekvat preventivmedel användas.
- Mental oförmåga, ovilja eller språksvårigheter som medför svårighet att förstå innebörden av att delta i studien.
- Ändring i dosering av immunsuppresiv behandling (azathioprin, 6-mercaptopurin eller metotrexat senare än 3 månader före besök 1; monoklonala antikroppar senare än 8 veckor före besök 1.
- Behandling eller sjukdom som enligt prövaren kan påverka behandlingen eller studieresultatet.

## 6.4 ”Withdrawal criteria”

- Graviditet eller önskan om att bli gravid.
- Patienten kan avsluta sitt deltagande i studien när som helst utan att detta medför några som helst konsekvenser for hans/hennes fortsatta behandling.
- Läkare/sponsor kan när som helst avsluta studien pga. biverkning eller att patienten inte följer procedurerna i studieprotokollet*.*

# 7 Tidsplanering

Planerad rekryteringsperiod: 18 månader

Planerad inklusion av första patient: QI 2006

Planerad tidpunkt för ”Final” studierapport: QIV 2007

# 8 Metodbeskrivning

## 8.1 Tillvägagångssätt

Patienter rekryteras från mottagningen på medicinsk gastroenterologi och hepatologi, UMAS i samband med sedan tidigare planerade återbesök, d v s rekryteringen sker ej från tidigare eller pågående studier.

Patienter som som väljer att avbryta studien kommer att istället följas upp och erhålla medicinsk eller kirurgisk behandling enligt gängse rutiner vid sektionen.

Samtliga patienter som screenas inför studien kommer att registreras i patientens medicinska journal samt av prövaren förd ”screening log”. Samtliga patienter som inkluderas i studien kommer att registreras i patientens medicinska journal samt av prövaren förd ”enrolment log”.

Patienten kommer att tilldelas ett ID kort som talar om att personen deltar i en studie, vem som är ansvarig och vem som kan kontaktas om så skulle behövas (namn och telefonnummer till ansvarig prövare kommer att finnas på detta kort).

### Besök 1 (Screening, v. 0)

I samband med mottagningsbesöket erhåller patienten både skriftlig och muntlig information.

Innan några studierelaterade åtgärder utförs på patienten har personen skrivit under ett informerat samtycke (”Informed Consent”).

Patienten får ett löpnummer som identifikation.

Sjukdomshistoria och aktuell medicinering kontrolleras.

Inklusion- och exklusionskriterier kontrolleras.

Puls, blodtryck och vikt kontrolleras.

Blodprover tas.

Faecesodlingar tas (salmonella, shigella, yersinia, campfylobacter samt clostridium difficle.

Patienten erhåller dagbok för att till nästa besök kunna registrera aktivitets index, CDAI.

Besök 2 ska infalla 8-15 dagar efter besök 1.

### Besök 2 (Enrollment, v.2)

Patienten lämnar i samband med detta besök avföringsprov för analys av calprotektin.

Inklusion- och exklusionskriterier kontrolleras.

Kontroll av oönskade händelser (Advers Events).

Patienten erhåller studieläkemedlet, atorvastatin, som delas ut på kliniken.

### 8.1.3 Besök 3 (v.6)

Blodprov tas.

CDAI kontrolleras.

Kontroll av oönskade händelser (Advers Events).

### 8.1.4 Besök 4 (v.15)

Blodprov tas.

CDAI kontrolleras.

Kontroll av oönskade händelser (Advers Events).

Patienten lämnar i samband med detta besök avföringsprov för analys av calprotektin.

Patienten avslutar behandling med studieläkemedlet.

Puls, blodtryck och vikt kontrolleras.

### 8.1.5 Besök 5 (Avslutande besök, v.23)

Blodprov tas.

CDAI kontrolleras.

Puls, blodtryck och vikt kontrolleras.

Kontroll av oönskade händelser (Advers Events).

Patienten återgår till ordinarie mottagningsuppföljning.

## Effektvariabler

### Blodprov

De blodprov som kommer att tas vecka 0, 6, 15 samt 23 är: Hb, hematokrit, leukocyter, trombocyter, CRP, albumin, kreatinin, ASAT, ALAT, kreatininkinas, totalkolesterol, LDL, HDL och triglycerider.

Blodprov tas även till flödescytometri för att immunfenotypa perifera monocyter vid dessa tillfällen. Analyser sker i samband med provtagning, biobankslagen tillämpas.

### Calprotektin

Calprotektin används som ett mått för att bedöma inflammationsgraden i tarmkanalen. Patienten lämnar avföringsprov för analys vecka 2 och 15. biobankslagen tillämpas.

### Crohn’s Disease Activity Index (CDAI)

Kliniskt index för att bedöma aktivitet av sjukdomen, se bifogad patientdagbok samt ref. [10]. Kontrolleras vecka 2, 6,15 och 23.

## Säkerhetsvariabler

### Oönskade händelser (Adverse Events=AE)

Patienter med Crohns sjukdom har regelbunden kontakt med gastromottagningen och kan vid eventuell oönskad händelse, när som under kontorstid ringa sjuksköterska som kontaktar ansvarig läkare. Detta registreras i patientens medicinska journal samt i CRF som AE.

Perioden för uppföljning av oönskade händelser sträcker sig från studiestart till studiens avslutande.

# Samtidig medicinering

Samtidig medicinering kommer att följas under studien och alla förändringar av dessa skrivas in i patientens CRF. Detta innebär den medicinering som tas under studien, förutom studieläkemedlet. Eventuella ändringar kan få inflytande på patientens lämplighet att fortsätta sitt deltagande i studien.

Med hänsyn till den relativt höga dosen atorvastatin och risken för interaktionsberoende biverkningar, särskilt myosit/rhabdomyolys bör samtidig medicinering med följande läkemedel undvikas: ciklosporin, erytromycin, klaritromycin, itrakonazol, ketokonazol, nefazodon, nikotinsyra, gemfibrozil, andra fibrater eller HIV-proteashämmare.

# 10 Samtidiga sjukdomar

Alla sjukdomar som patienten har vid studiestarten ska skrivas in i patientens CRF. Alla sjukdomar/symtom patienten får under studiens pågående, ska hanteras som oönskade händelser (AE=”Adverse Events”). Se sektion 12.

# 11 Studiematerial

## 11.1 Läkemedel

Studieläkemedlet är det registrerade läkemedlet atorvastatin (Lipitor®), ATC-kod C10A A05. Filmdragerad tablett. Tillverkare Pfizer.

## Dosering/behandlingsregim

Studieläkemedlet tas av patienten i hemmet per os, en gång per dag. Dosen är 80 mg dagligen. Dosen kan ges vid valfri tidpunkt på dagen med eller utan samtidigt intag av föda. Doseringen är fast under studien. Behandlingstid 3 månader.

## Förpackning och förvaring av studieläkemedel

Tabletterna är blisterförpackade med en hållbarhet på 3 år.

Inga särskilda förvaringsanvisningar. Studieläkemedlen förvaras i rumstemperatur.

## Läkemedelshantering (”Drug Accountability”)

Studieläkemedlet ska lämnas ut till patienten vid klinikbesöket vecka 2. Ansvarig prövare tillhandahåller studieläkemedlet.

Prövare eller forskningssjuksköterska som hanterar studieläkemedel ska föra in antal/mängd utlämnat respektive återlämnat läkemedel på avsedd sida i CRF.

Ansvarig monitor eller delegerad person, kontrollerar att läkemedelshanteringen sköts optimalt samt ombesörjer att destruktion av överblivna och returnerade läkemedel hanteras efter överenskommelse enligt tecknat Apoteksavtal.

## Randomisering och blindning

Studien är en öppen pilot studie utan kontrollgrupp.

## 11.6 ”Compliance”

Patienten måste ha tagit minst 75 % av studieläkemedlet för att kunna räknas som ”compliant” och därmed kunna ingå i per-protokoll analysen. Se även statistikavsnittet.

# ”Adverse Events” (oönskade händelser)

## 12.1 Oönskade händelser (AE)

**AE** = ”Adverse Events” = oönskade händelser.

”Adverse Event” är alla oönskade händelser som inträffar under en studie, oavsett om de anses ha ett samband med studieläkemedlet eller ej.

Samtidiga sjukdomar som rapporterats vid studiestart kommer inte att rapporteras som ”oönskade händelser” (AEs). Dock kommer en eventuell förändring av sjukdomstillståndet som inträffar under studien rapporteras som en AE.

## ”Serious Adverse Events”

**SAE** = ”Serious Adverse Event” = allvarlig oönskad händelse.

En allvarlig oönskad händelse eller effekt som oavsett dos resulterar i något av följande tillstånd;

- Dödfall.
- Livshotande situation.
- Kräver sjukhusvård eller förlänger ett redan påbörjat vårdtillfälle på sjukhus.
- Förorsakar ett varaktigt eller betydelsefullt handikapp eller funktionsnedsättning.
- Utgör en medfödd missbildning eller medfödd defekt.

## “Suspected Unexpected Serious Adverse Reaction”

**SUSAR** = “Suspected, Unexpected Serious Adverse Reaction” = misstänkt oönskad allvarlig reaktion.

En misstänkt oönskad allvarlig svarsreaktion på ett försöksläkemedel som oavsett dos resulterar i något av ovan nämnda tillstånd (12.2, SAE). Dessa kommer att rapporteras till berörda myndigheter enligt gällande föreskrifter i LVFS 2003:6.

## 12.4 Definitioner

### 12.4.1 Svårighetsgrad

Följande gradering används för att gradera svårighetsgraden på en oönskad händelse;

- **Mild**: kortvarig/övergående symtom som inte påverkar patienten/försökspersonen dagliga aktiviteter.
- **Moderat**: påtaglig/uppenbara symtom som till en del påverkar patienten/försökspersonen dagliga aktiviteter.
- **Svår**: symtom som väsentligt påverkar patienten/försökspersonen dagliga aktiviteter.

### 12.4.2 Relation till studieläkemedel

Nedanstående definition kommer att användas för att bedöma relationen mellan en oönskad händelse och studieläkemedlet.

- **Sannolik:** god grund och tillräcklig dokumentation för att anta att en relation mellan symtom och studieläkemedel föreligger.
- **Möjlig:** ett möjligt orsakssamband är tänkbart och kan inte bortses från.
- **Osannolik:** symtom som troligen inte har något samband med studieläkemedlet.

## 12.5 Rapportering av oönskade händelser

- Patienten kommer att tillfrågas vid varje kontakt med prövare/sjuksköterska om han/hon haft några oväntade händelser (AE) sedan förra besöket/kontakten.
- Oönskade händelser rapporteras oavsett om de har relation till behandlingen eller inte.
- Alla rapporterade ”oönskade händelser” som inte är återställda vid avslutande av studieläkemedlet följs upp in samband med avslutningsbesöket vecka 23.
- Om en patient som deltar i denna läkemedelsstudie blir gravid, följs personen upp tills efter förlossningen ägt rum. Eventuella komplikationer under graviditet rapporteras som en AE. Om fostret/barnet har någon medfödd missbildning rapporteras detta som en allvarlig oönskad händelse (SAE).

### 12.5.1 Rapportering av SAE - allvarliga oönskade händelser

De allvarliga oönskade händelserna (SAEs) som inträffar under studien rapporteras till LV och REPN.

### 12.5.2 Rapportering av SUSAR – misstänkta allvarliga oönskade reaktioner

- En **misstänkt, allvarlig oväntad svarsreaktion** på försöksläkemedlet (SUSAR), som är **dödlig eller livshotande** **rapporteras** till LV och REPN **inom 7 dagar** efter ansvarig prövare fått kännedom om händelsen. Komplettering av rapporten skickas till myndigheterna inom max 15 dagar.
- En **misstänkt, allvarlig oväntad svarsreaktion** på försöksläkemedlet (SUSAR), som **inte** är dödlig eller livshotande **rapporteras** till LV och REPN **inom 15 dagar** efter ansvarig prövare fått kännedom om händelsen. Komplettering av rapporten skickas till myndigheterna snarast.

# Patientformulär (CRF= ”Case Report Form”)

Följande gäller;

- Att alla frågor i patientformuläret måste besvaras.
- Om någon fråga inte kan besvaras, fylls detta fält i med; ND=”Not done”, NA=”Not applicable”, NK=”Not known”.
- Eventuella korrektioner görs genom att stryka över det felaktiga samt datera och signera ändringen och skriv den korrekta informationen bredvid.
- De patientdata som finns i patientjournalen måste överensstämma med de data som är skrivna i patientens patientformulär (CRF).

## Andra patientformulär

Patienterna får en dagbok där antal avföringar, magsmärtor, allmänt välbefinnande, feber > 38, ev. läkemedel mot diarré, ledbesvär, ögoninflammation, hudutslag, annalfistel eller fissur samt ev. annan fistel dokumenteras. Detta dokumenteras dagligen av patienterna veckan innan besök i vecka 2, 6, 15 samt 23. Dagbok förs för att kunna räkna ut patientens CDAI.

# Monitorering

Innan någon studierelaterad åtgärd/undersökning kan påbörjas ska patient/försöksperson informeras och samtycke inhämtas om att monitor samt eventuell kontrollmyndighet får tillgång till patientjournal för att verifiera källdata. Verksamhetschefen ska ge sitt tillstånd till att en utomstående monitor får ta del av relevant patientdata i patientjournalerna. En sekretessförbindelse ska upprättas mellan verksamhetschefen som är ansvarig för patientjournalerna och monitor.

Monitorering av studien kommer att genomföras av en oberoende monitor från RSKC, Region Skånes Kompetens Centrum för klinisk forskning, Universitetssjukhuset, 221 85 Lund.

Monitor kommer att säkerställa följande;

- Att protokollet följs.
- Att rekrytering och inkludering av patienter i studien går enligt studieplanen.
- Att korrekt data samlas in i för studien framtagna ”Case Report Form” – CRF.
- Att oönskade händelser rapporteras enligt LVFS 2003:6.
- Att hantering av läkemedel sker enligt protokollet samt föreskrifter för klinisk prövning
- Att tillgång till originaldata och studierelaterade dokument finns tillgängliga samt att all dokumentation sparas/arkiveras.
- Att resurser finns tillgängliga för genomförande av studiens alla delar.

Ansvarig prövare ska se till att monitor har tillgång till CRF, patientjournal samt original av laboratoriedata etc. för att kontrollera källdata relevanta för studien, utan att äventyra patientens integritet.

Ansvarig prövare är också medveten om att inspektion från myndighet kan komma att genomföras.

## Källdata

Data i patientformuläret (CRF) kontrolleras enligt överenskommelse. De patientdata som finns i patientjournalen måste överensstämma med de data som är skrivna i patientens/försökspersonens patientformulär (CRF).

# Datainsamling och databearbetning

Patienterna identifieras med sina initialer samt ett löpnummer. Patienternas fulla identitet kopplat till löpnummer i studien kommer att finnas på en patientidentifikationslista.

Patientdata kommer att bearbetas och hanteras av ansvarig prövare på Universitetssjukhuset MAS, Malmö .

Patientdata kommer att bearbetas/hanteras efter studien har avslutats.

# Etiska överväganden

Studien utförs i överensstämmelse med gällande Helsingforsdeklaration.

Fördelen för patienter med Crohns sjukdom är möjligheten att hitta en effektiv behandling som är förenat med få biverkningar. Läkemedlet, atorvastatin, som kommer att användas är oftast förenat med biverkningar av mild och övergående natur. De vanligast förekommande biverkningarna är huvudsakligen gastrointestinala, såsom obstipation, flatulens, dyspepsi, buksmärtor och går vanligen över vid fortsatt behandling. Serumnivåer av transaminaser och kreatininkinas kommer att följas under studien för att detektera eventuell muskelpåverkan.

Upptäckten och introduktionen av potenta, immunomodulerande alternativ som saknar oönskade biverkningar, skulle vara ett stort kliniskt genombrott i behandlingen av denna svårbehandlade patientgrupp. Trots att vi får lov att vara försiktiga att extrapolera våra tidigare resultat [4-6] till en bredare klinisk situation, indikerar de om en ny möjlighet att se på behandlingen av kronisk inflammation som vidmakthålls via monocytrekrytering, genom att modulera den intracellulära lipidhomeostasen. Därutöver finns det en stor klinisk dokumentation av HMG-CoA reduktas hämmare, då de är brett använda och har visat sig ha få sidoeffekter, vilket gör dem till lämpliga kandidater för underhållsbehandling.

## Medgivande

I studien kommer samtyckesprocessen att utföras enligt ICH GCP guideline och Helsingfors deklarationen, dvs. patienten måste ge ett skriftligt medgivande till att delta i studien, innan någon studierelaterad åtgärd utförs på patienten. En kopia av patientinformationen samt av det signerade medgivandet kommer att lämnas till patienten.

Ansvarig prövare kommer att ge både muntlig och skriftlig information till den tilltänkta patienten angående vad ett eventuellt deltagande i studien innebär.

## Regional etik prövningsnämnd (REPN)

Inklusion av patienter i studien startar när skriftligt godkännande från den Regionala etikprövningsnämnden (REPN) samt tillstånd från Läkemedelsverket (LV) finns tillgängligt.

All korrespondens med REPN arkiveras i den för studien avsedda prövarpärmen.

Det är viktigt att datum och versionsnummer finns tydligt angivet på alla dokument som skickas till REPN för etisk prövning. Denna information bör även stå på följebrevet till ansökan till REPN.

# 17 Läkemedelsverket (LV)

Inklusion av patienter i studien startar när skriftligt godkännande från den Regionala Etikprövningsnämnden (REPN) samt tillstånd från Läkemedelsverket (LV) finns tillgängligt.

All korrespondens med LV arkiveras i den för studien avsedda prövarpärmen.

# 18 Avbrytande av studien

Prövare/sponsor kan när som helst avbryta studien. Om så blir fallet, ska prövaren direkt informera alla som deltar i studien. Vidare ska prövaren skriftligen informera LV och REPN inom 15 dagar på avsedd blankett.

# 19 Statistiska överväganden

Studien är planerad som en "intention to treat"-analys och resultaten kommer att analyseras med Wilcoxons parade test. Då det är en pilotstudie tillämpas ej någon "power"-beräkning. I en pilotstudie rekomemenderas analysdata från minst 8 patienter, men då vi räknar med ett visst bortfall har vi valt att inkludera 12 patienter.

Resultat från patienter som eventuellt avbryter studien p g a biverkningar kommer att tas med i analysen. Eventuell avsaknad av patientdata kommer att dokumenteras i CRF, dock bedöms detta som ett mindre problem då studien är så pass liten och utförs enbart på ett centra.

Ingen interimsanalys är planerad.

För att gå vidare med en större nationell studie vill vi gärna uppnå en skillnad före och under behandling på minst 30% på effektmåtten som bedömer inflammationsgraden i blodet.

# 20 Patientförsäkring

Patienter/försökspersoner är försäkrade genom Läkemedelsförsäkringen och Patientskadelagen.

# 21 Rapport och publicering

Studiedata ägs av forskningshuvudmannen.

Bearbetning av resultaten kommer att ske under 2007.

Studien kommer att redovisas till internationella medicinska tidskrifter under 2007, i förhoppning om publicering början av 2008.

Författare till studien kommer att vara Olof Grip, Anders Bredberg, Stefan Lindgren och Sabina Janciauskiene, samtliga vid Universitetssjukhuset MAS, Malmö.

# 22 Förvaring/Arkivering

Ansvarig prövare/sponsor har en sk prövarpärm med relevant innehåll enligt studien och ICH kapitel 8 ”Essential documents”. Ansvarig prövare/sponsor kommer att förvara CRF, patientidentifikationslista, original av patientinformationer och inhämtade samtycke för studien oåtkomligt för obehöriga, men så att patienter i studien kan identifieras av de ansvariga för studien.

Arkivering av studiedokument, samt källdata kommer att arkiveras minst 10 år efter studierapporten är skriven och inskickad till Läkemedelsverket.

# Referenslista

1. Mashimo H, Wu DC, Podolsky DK, Fishman MC. *Science*, **1996**;*274*:262.
2. Takemoto M, Liao JK. *Arterioscler. Thromb. Vasc. Biol*., **2001**;*21*:1712.
3. Liao JK. *J. Clin. Invest*., **2002**;*110*:285.
4. Grip O, Janciauskiene S, Lindgren S. *Inflamm Bowel Dis*, **2004**; *10*:193.

1. Grip O, Janciauskiene S, Lindgren S. *Eur. J. Pharmacol*., **2000**;*410*:83.
2. Grip O, Janciauskiene S, Lindgren S. *Inflamm. res*., **2002**; *51*: 58.
3. McCarey DW, McInnes IB, Madhok R, Hampson R, Scherbakov O, Ford I, Capell HA, Sattar N. *Lancet,* **2004**;363:2015.
4. Vollmer T, Key L, Durkalski V, Tyor W, Corboy J, Markovic-Plese S, Preiningerova J, Rizzo M, Singh I. *Lancet,* **2004**;363:1607.
5. Bernini F, Poli A, Paoletti R. *Cardiovasc Drugs Ther* , **2001**;15:211.
6. Best WR, Becktel JM, Singleton JW, et al. *Gastroenterology,* **1976**;70:439.

Atorvastatin vid Crohns sjukdom

Patient-ID:___________ Initialer:___________

##### DAGBOK

Här kan du fylla i de symptom du upplever under veckan:

1. Avföring registreras i tabellen strax efter att du har gått på toaletten.

2, 3. Graden av magsmärtor och allmänt välbefinnande registreras var dag innan sänggående och ska beskriva den genomsnittliga känslan av magsmärta eller välbefinnande under dagen.

4. Om du har feber skriver du in den uppmätta temperaturen.

| Datum |  |  |  |  |  |  |  |
| --- | --- | --- | --- | --- | --- | --- | --- |
| 1. Antal avföringar |  |  |  |  |  |  |  |
| 1. Magsmärtor   0=inga  1=milda  2=måttliga  3=svåra |  |  |  |  |  |  |  |
| 1. Allmänt välbefinnande   0=allmänt bra  1=lite under medel  2=dålig  3=mycket dålig  4=förfärligt dålig |  |  |  |  |  |  |  |
| 4. Feber över 37,8 (munnen) eller 38,3 (rektum) |  |  |  |  |  |  |  |

Aktuella mediciner:____________________________________________________

____________________________________________________

Tar du något läkemedel mot diarré? Nej  Ja 

Om ja, vilka?____________________________________________________

Övriga besvär den sista veckan:

|  | Ja | Nej |
| --- | --- | --- |
| Ledbesvär |  |  |
| Ögoninflammation |  |  |
| Hudutslag |  |  |
| Analfistel eller fissur |  |  |
| Annan fistel |  |  |

# Patientinformation

Atorvastatin (Lipitor®) vid måttligt aktiv Crohns sjukdom

Härmed tillfrågas du om du vill delta i en studie avseende behandling av Crohns sjukdom med atorvastatin. Ditt deltagande är helt frivilligt och du kan när som helst avbryta ditt deltagande utan att lämna särskilda skäl till detta. Du kommer i så fall att erbjudas gängse behandling med annan typ av terapi (ex.vis andra läkemedel alternativt operation.

Crohns sjukdom är en kronisk tarminflammation där orsakerna till att man drabbas är okända. Skadorna på tarmslemhinnan bedöms dock till stor del bero på negativa effekter av de aktiverade vita blodkropparna som ansamlas i tarmvävnaden i samband med den pågående inflammationen.

Etablerade läkemedel mot Crohns sjukdom, som t. ex. kortison, avser att hämma de aktiverade vita blodkropparna, men är i många fall otillräckliga och kan ibland ge upphov till oönskade biverkningar.

Det registrerade läkemedlet av atorvastatin heter Lipitor® och används för att sänka blodfetter, men flera studier tyder på att det även har egenskaper som minskar inflammationen genom att påverka ansamling och aktivering av vita blodkroppar. Atorvastatin är bland de mest förskrivna läkemedel i världen, och stor erfarenhet finns när det gäller biverkningar och säkerhet.

**Syfte**

Syftet med denna studie är att se om atorvastatin kan påverka inflammationen gynnsamt vid Crohns sjukdom i hopp om att hitta behandlings alternativ som är förenat med få biverkningar.

**Hur studien går till**

Du kommer att erhålla en tablett Lipitor® 80 mg dagligen som tillägg till din övriga medicinering. Studien varar i 6 månader. För att utvärdera om du har någon effekt av behandlingen kommer blodprov tas innan och under behandling, sammanlagt kommer ca 100 ml blod att tas under studien. Om du inte har gjort en koloskopi inom de senaste 3 månaderna kommer du genomgå en sådan för att bedöma inflammationen i tarmen innan samt ytterligare en undersökning efter 3 månader. Effekten kommer även att utvärderas med hjälp av ett standardiserat formulär, CDAI, som avser dina symptom i form av buksmärtor, diarré, mm.

**Fördelar**

Fördelen för patienter med Crohns sjukdom är möjligheten att hitta en effektiv behandling som är förenat med få biverkningar.

**Nackdelar**

De vanligast förekommande biverkningarna är huvudsakligen förknippade till magtarmkanalen, såsom förstoppning, gasbildning, bukobehag, buksmärtor och går vanligen över vid fortsatt behandling. Behandlingen kan i enstaka fall ge en muskelpåverkan varför blodprov för detta kommer att följas under studien. Om du är kvinna i barnafödande ålder rekomenderas du använda prevmentivmedel då behandlingen kan passera över och eventuellt påverka foster.

**Personuppgiftsansvar**

Region Skåne är personuppgiftsansvarig. Alla resultat registreras konfidentiellt och behålles av ansvarig läkare för studien, dr Olof Grip. Parallellt sker sedvanlig dokumentation i din patientjournal i samband med mottagningsbesöken. Du kan vända dig till ansvarig läkare för studien om du önskar utdrag över de personuppgifter som finns registrerade på dig och ev. hjälp till rättelse.

**Försäkring**

Patienter som deltager i kliniska prövningar är skyddade av patientskadelagen och läkemedelsförsäkringen.

Studien är granskad och godkänd av regionala etikprövningsnämnden vid Lunds universitets samt Läkemedelsverket.

Önskas mer information går det bra att ta kontakt med ansvariga för studien:

Ansvarig läkare: Ansvarig sjuksköterska: Gastrosektionen, medicinkliniken

Olof Grip Mariette Bengtsson UMAS, Malmö, tel: 040-33 23 06

Informerat samtycke

Studien “Atorvastatin vid måttligt aktiv Crohns sjukdom”

Jag har informerats muntligt om studien och jag har läst den skriftliga informationen. Jag har fått svar på mina frågor och samtycker till att delta i studien. Jag vet att mitt deltagande är frivilligt och att jag kan när som helst utan förklaring avbryta mitt deltagande i studien, utan att det påverkar mitt fortsatta omhändertagande.

Jag har också fått information att de uppgifter som samlats in om mig i denna studie kommer att behandlas konfidentiellt, på ett sådant sätt att min identitet inte kommer att röjas för obehöriga.

Jag tillåter att de uppgifter som samlats in i studien kan utlämnas av ansvarig läkare till medicinska kontrollmyndigheter i olika länder, regional etikprövningsnämnd eller till personer som arbetar med studien och att uppgifterna eventuellt kan komma att överföras till tredje land. Allt under förutsättning att sedvanlig sekretess upprätthålls.

Jag har även informerats om min rättighet att få registerutdrag en gång per år, över de uppgifter och eventuellt provmaterial som är insamlat. Jag har dessutom informerats om rättigheten att få provmaterial förstört och möjligheten att få felaktiga uppgifter tillrättalagda eller borttagna ur registret.

…………………………………………………. ……………………………………………….

(namnteckning, försöksperson) (namnteckning, ansvarig läkare)

…………………………………………………. ……………………………………………….

(namnförtydligande) (namnförtydligande)

…………………………………………………. ……………………………………………….

(ort, datum) (ort, datum)

Jag har informerats om och samtyckt till att en oberoende granskare (monitor) och läkemedelsmyndighet vid behov får jämföra de i studien rapporterade uppgifterna med de som finns i min patientjournal. Detta får ske under förbehåll att den information som därvid bli tillgänglig inte förs vidare.

…………………………………………………. ……………………………………………….

(namnteckning, försöksperson) (ort, datum)

**Namn och adresslista**

###### **Forskningshuvudman**

Region Skåne

Företrädare

Marek Wroblewski, verksamhetschef

Medicinkliniken, universitetssjukhuset MAS

205 02 Malmö

###### **Ansvarig prövare, tillika sponsor**

Namn: Olof Grip, med.dr., specialistläkare

Adress: Sektionen för gastroenterologi och hepatologi, medicinkliniken, UMAS, 205 02 Malmö

E-postadress: olof.grip@med.lu.se

Telefon: 040/33 61 56

##### Andra medverkande

Namn: Anders Bredberg, docent, överläkare
Adress: Medicinsk mikrobiologi, Hs 33, UMAS, 205 02 Malmö

E-postadress: Anders.Bredberg@mikrobiol.mas.lu.se

Tel: 040-337414

Namn: Stefan Lindgren, professor, överläkare

Adress: Sektionen för gastroenterologi och hepatologi, medicinkliniken, UMAS, 205 02 Malmö

E-postadress: stefan.lindgren@medforsk.mas.lu.se
Tel: 040-332306

Namn: Daniel Klintman, med. dr., leg. läkare

Adress: Sektionen för gastroenterologi och hepatologi, medicinkliniken, UMAS, 205 02 Malmö

E-postadress: daniel.klintman@medforsk.mas.lu.se

Tel: 040-33 10 00

Namn: Sabina Janciauskiene, docent, forskare

Adress: Wallenberglaboratoriet, plan 2, UMAS, 205 02 Malmö

E-postadress: sabina.janciauskiene @medforsk.mas.lu.se

Tel: 040-33 14 14

##### Oberoende monitor

RSKC, Region Skånes Kompetens Centrum för klinisk forskning, Universitetssjukhuset, 221 85 Lund.

Samtycke till att spara studierelaterat provmaterial i Biobank

Studien ”Atorvastatin vid måttligt aktiv Crohns sjukdom”

Jag samtycker till att blodprov får bevaras i en s.k. biobank vid medicinska kliniken, Universitetssjukhuset MAS. Jag har fått information om att provmaterialet undersöks för att försöka klarlägga om Lipitor® kan ha en antiinflammatorisk effekt vid Crohns sjukdom.

Jag är medveten om att provmaterialet inte kommer att användas till andra syften än ovan nämnda utan att jag informerats och givit samtycke till det nya ändamålet.

Jag är också medveten om att jag på begäran hos ansvarig kontaktperson för studien eller vid kontakt med det regionala biobankskontoret kan få blodprovet förstört.

………………………………………………….

(namnteckning, försöksperson)

………………………………………………….

(namnförtydligande)

………………………………………………….

(ort, datum)

Atorvastatin vid måttligt aktiv Crohns sjukdom

Sammanfattning av studieprotokollet

EudraCT nr:2005-000496-17

Studien planeras som en öppen pilotstudie för att inhämta information avseende effekten av atorvastatin vid Crohns sjukdom. Studien är lokaliserad till ett centra med en forskningshuvudman. Syftet med studien är att efterforska huruvida atorvastatin (Lipitor®) har en kliniskt immunmodulerande effekt på patienter med måttligt aktiv Crohns sjukdom. Tidigare studier saknas. Patienter med Crohns sjukdom saknar bra underhållsbehandling, varför det är av stor vikt att få fram alternativ till denna svårbehandlade patientgrupp.

Studieläkemedlet är välbeprövat vid hyperkolesterolemi och en av de mest förskrivna läkemedel globalt sett med relativt beskedliga biverkningar. Publicerad data finns att läkemedlet har antiinflammatoriska egenskaper på patienter med annan kronisk inflammation såsom vid reumatoid artrit och multipel skleros. Studien vill göras för att kunna motivera en senare placebokontrollerad nationell studie.

Till 12 patienter med måttligt aktiv Crohns sjukdom erbjuds tilläggsbehandling med 80 mg atorvastatin dagligen under 3 månader. Patienterna rekryteras från mottagningen för medicinsk gastroenterologi och hepatologi, medicinkliniken, UMAS.

Inflammationsmarkörer i blodet kontrolleras innan behandlingsstart samt under behandling. De markörer som kommer att bedömas är CRP, MCP-1, TNF-alfa samt immunfenotypen av cirkulerande monocyter med hjälp av flödescytometri. Den kliniska aktiviteten kommer att bedömas med hjälp av ett aktivitetsindex, CDAI. Inflammationen i tarmen kommer att bedömas med hjälp av analys av kalprotektin i faeces innan och efter behandling.

I studien ingår 5 klinikbesök där patienten för dagbok veckan innan vardera besök. Den totala studietiden är 23 veckor. Studien kommer att utföras under 2006-2007.

Studien är obunden finansiering från läkemedelsindustrin.
